# Face-to-Face Lying: Gender and Motivation to Deceive

**DOI:** 10.3389/fpsyg.2022.820923

**Published:** 2022-03-22

**Authors:** Eitan Elaad, Ye’ela Gonen-Gal

**Affiliations:** Department of Psychology, Ariel University, Ariel, Israel

**Keywords:** lying, detection of deception, gender, motivation, self-assessed abilities, information processing style

## Abstract

Two studies examined gender differences in lying when the truth-telling bias prevailed (study 1) and when inspiring lying and disbelief (study 2). The first study used 156 community participants (91 women) in pairs. First, participants completed the Narcissistic Personality Inventory, the Lie- and Truth Ability Assessment Scale (LTAAS), and the Rational-Experiential Inventory. Then, they participated in a deception game where they performed as senders and receivers of true and false communications. Their goal was to retain as many points as possible according to a payoff matrix that specified the reward they would gain for any possible outcome. Results indicated that men lied more and were more successful lie-tellers than women. In addition, men believed the sender less than women but were not more successful detectors of lies and truths. Higher perceived lie-telling ability, narcissistic features, and experiential thinking style explained men’s performance. The second study used 100 volunteers (40 women) who underwent the same procedure. However, the payoff matrix encouraged lying and disbelieving. Results showed again that men lied more than women. As to performance, men were more successful lie detectors than women, but there was no truth detection difference. Women did not differ in their success in telling and detecting lies and truths. The inconsistent gender differences in production and detection lies and truths dictate caution in interpreting them.

## Introduction

The current view about frequent lying is that not many people lie frequently, and most reported not lying in the previous 24 h (Serota et al., 2010; Halevy et al., 2014; Serota and Levine, 2015; Daiku et al., 2021). For example, Serota et al. (2010) asked 1,000 adults to report the number of lies they told in 24 h. Five percent of the responders were responsible for telling almost half of all lies, and 60% reported telling no lies. A few prolific liars told most reported lies.

The different lying tendency implies that in any given situation, some people lie while others refrain from lying. Therefore, we designed the present study to explore further these differences in a sender-receiver deception game focusing on the inconsistent gender differences in lying and in lie detection under situations that encourage truth-telling and believing (first experiment) and under conditions that promote lying and disbelief (second experiment).

We also made predictions of relevant individual features such as perceived lying and lie detection abilities, narcissistic marks, and rational/experiential information processing style on deception and believing in the game. The present study is the first to study the influence of rationale and experiential processing styles in the context of interactive lying.

The current study used a sender-receiver deception game in which senders hid either a white or a black ball in their fists and tried to convince receivers that they were hiding a white ball. Receivers had to decide whether the sender was telling the truths or lying. After 10 trials, participants changed roles, and the game continued for another 10 periods. The participant’s goal was to win the game by collecting more points than the other player. Points were allocated according to a payoff matrix that specified each player’s points for any possible result. The decision to select a white or a black ball at any trial was left entirely to the sender’s discretion, given that they chose a white ball and a black ball at least once.

## Experiment 1

### Gender Differences in Frequent Lying

Gender differences in lying have been extensively studied with mixed results (e.g., Dreber and Johannesson, 2008; Childs, 2012). Some accounts suggested that when lies benefit the liar at the expense of another person, men tend to lie more than women (e.g., Friesen and Gangadharan, 2012). However, some other studies failed to report such differences (e.g., Sweeney and Ceci, 2014). A meta-analysis on honesty (Gerlach et al., 2019), based on 380 experiments that recorded gender differences in lying, indicated that men were 4% more deceptive than women.

Another meta-analysis on gender differences in lying (Capraro, 2018) distinguished between black lies (that benefit the liar at the cost of another person), altruistic lies (lies that benefit another person at the expense of the liar), and Pareto white lies (that benefit both the liar and another person). Capraro reported the results of 65 experimental treatments and 8,728 observations and found that men are significantly more likely than women to tell black lies and altruistic white lies. However, results were inconclusive concerning Pareto white lies.

Black lies are the focus of the present study. We expect men to lie more than women in a sender-receiver game. A meta-analysis summarizing results from widely used personality inventories conducted between 1940 and 1992 supports this view. The meta-analysis showed that women scored slightly but consistently higher on trust (Feingold, 1994). Trust reflects a belief in the honesty and positive intentions of others. Women reported telling fewer lies than men and were less ambitious and less skilled in subtle, diplomatic persuasion (Kashy and DePaulo, 1996). It follows that women are more sensitive than men to honesty and therefore lie less.

Research on gender differences in believability varies. Buchan et al. (2008) indicated that women felt obligated to trust more than men, but men have more faith than women. Haselhuhn et al. (2015) examined how gender moderate responses to trust violation. They reported that women are less likely to lose trust in a transgressor than men. The desire to maintain relationships explains the faith of women.

Other variables that were not yet given the proper attention might moderate the gender effect. Men sensitivity to monetary gains and losses may be an example. Eckel and Grossman (2001) showed that women accept lower offers than men in the ultimatum game where two players share money. One player offers the other player part of the initial amount, and the recipient accepts or rejects the offer. In the dictator game, a version of the ultimatum game where the recipient must accept any monetary offer, men are more selfish than women (Croson and Gneezy, 2009).

Further, men tend to donate less than women to charity (Piper and Schnepf, 2008; De Wit and Bekkers, 2016). It follows that men are more focused on money than women, who are more sensitive to the social context of the experiment. The different focus explains why men lie more than women when lying leads to a monetary profit.

Most previous studies on gender differences in deception games offered a monetary benefit to motivate participant’s actions (e.g., Gneezy, 2005; Dreber and Johannesson, 2008; Gneezy et al., 2013). Based on earlier experience with game points (Elaad et al., 2020), we used game points instead of cash to control a possible monetary influence on the lying behavior of men and women. We assume that game points are no less effective than a small amount of money to motivate action in the deception process. Furthermore, using points generates a pure winning experience that money does not contaminate.

In addition, most deception studies were conducted in samples of students (Gerlach et al., 2019), while the present study sampled participants from the local community. The difference is significant because results suggested that students behave more dishonestly than a broader sample of the population (e.g., Abeler et al., 2014; Fosgaard et al., 2018). Still, Gunia et al. (2014) reported no difference. Gerlach et al. (2019) explained that students are typically younger than the general population, and the young age contributes to dishonest actions. Furthermore, students have cognitive abilities essential to dishonest behavior.

Capraro (2018) meta-analysis did not consider the gender of the target person to whom the liar directs the lies. The target’s gender is relevant because earlier accounts showed that men contributed more and volunteered more time to charity in the presence of a women audience (Van Vugt and Iredale, 2013). In the context of lying, we may suggest that chivalry drives men to be more honest with female targets than male targets, which evoke competition. Gender solidarity urges women to lie less to female targets than male targets (Eckel and Grossman, 2001).

### Gender Differences in Lying and Lie-Detection Efficiency

Past research on lie-detection suggested that most lies go undetected (e.g., Bond and DePaulo, 2006). Accordingly, Sweeney and Ceci (2014) reported no gender differences in detecting lies. Burgoon et al. (2006) discussed gender differences in verbal and non-verbal behavioral cues and concluded that women tend to smile and gaze more and be more pleasant than men. The lovely appearance may help in creating a credible image. In contrast, women are more expressive and less relaxed than men, which people associate with nervousness and insincerity. It is conceivable to suggest that gender differences in efficient lying and lie detection may be minor and inconsistent. Hence, we will find no gender differences in lying and lie-detection efficiency.

### Preferring the Truth

Acting sincerely and genuinely is the preferred behavior, and therefore people lie surprisingly little. Prevalence, to tell the truth, was found in a meta-analysis by Abeler et al. (2019), who combined data from 90 experimental studies that used the die rolling paradigm of Fischbacher and Föllmi-Heusi (2013) (FFH), where participants decide autonomously and anonymously about their lying behavior. In this paradigm, participants privately observe the outcome of a random variable, report the product, and receive a monetary payoff proportional to their report. No one checks the accuracy of their account, and therefore, participants can easily misreport the obtained outcomes. Results indicated a tendency toward truth-telling. Abeler et al. (2019) concluded that people are motivated to look honest, which results in frequent truth-telling.

Nevertheless, truth preference is not limited to the FFH paradigm. Bradley (1988) allowed participants in a simulated polygraph test to choose between stealing a large sum of money and lying in the subsequent polygraph test or receiving a smaller sum as payment for participation in the experiment and telling the truth in the polygraph test. Bradley informed the participants that they could keep the money if they were found truthful in the polygraph test. Findings showed that 56 of 76 participants preferred to tell the truth. Robinson et al. (1998) presented participants with a scenario indicating that they are applying for an ideal job for which they have the necessary qualifications except for their age. The age requirement in the advertisement averts 25, while participants were only 23 years old. Results indicated that participants preferred to tell the truth about their age. Elaad (2019) showed that people tended to select implausible truths rather than plausible lies when assigned to the role of innocent suspects in simulated police investigation scenarios where their task was to convince an interrogator of their innocence.

The costly nature of lying may explain the preference for truth-telling. Lying is harmful for moral or religious reasons, self-image concerns, social norms of honesty, or entails a cost in challenging the target’s authority (Abeler et al., 2019).

Therefore, we expect many participants to be biased toward truth-telling and select more white balls than black balls in the sender’s position. But, on the other hand, we expect participants to believe the message that the hidden ball is white in the receiver’s position.

Croson and Gneezy (2009) noted that men are more competitive than women. We suggest that men win the game by lying more often than the truth bias advises. The less competitive women will stick to the truth bias irrespective of their earned points.

### Personality and Individual Differences in Lying

#### Narcissism

Narcissism, along with Machiavellianism and psychopathy, is part of the Dark-Triad traits associated with perceived deception production ability but not with the ability to detect deception and the perceived ability to detect deception (Wissing and Reinhard, 2017, 2019). Narcissism is a multifaceted personality construct representing self-centering, dominance, and manipulative interpersonal orientation (Emmons, 1987). Narcissists have a sense of entitlement, insufficient empathy, and feelings of superiority. They are abusive and perceive others as a device for gratifying their needs. Narcissists require others to admire them and expect to receive privileged treatment. However, despite their impractical sense of grandiosity, their self-esteem is unstable and highly dependent on their social interactions. Narcissists may respond with anger and resentment when their self-image is threatened (Kohut, 1978; Raskin and Terry, 1988; Rhodewalt and Morf, 1998; Ostrowsky, 2010; Sadock and Sadock, 2015). Baumeister and Vohs (2001) suggested that narcissism may be considered an addiction to esteem. Narcissists tend to self-enhance desirable qualities such as creativeness, intelligence, and physical attractiveness (e.g., Gabriel et al., 1994; Grijalva and Zhang, 2016). Narcissists do not consider themselves more decent than others and do not value morality. Grijalva and Zhang (2016) suggested that narcissists view communal characteristics as a sign of softness and helplessness. Using the six-factor HEXACO model, narcissists score low on the honesty-humility dimension. Specifically, narcissists show low degrees of fairness, modesty, sincerity, and greed avoidance (Lee and Ashton, 2005; Muris et al., 2017). However, Sedikides et al. (2004) claimed that normal narcissism correlated with good psychological health.

Earlier studies found a positive association between narcissism and reported lying or immoral behavior in daily life situations (Oliveira and Levine, 2008; Baughman et al., 2014; Jonason et al., 2014; Azizli et al., 2016). Narcissistic people further believe that they are more efficient liars than the average person (Giammarco et al., 2013; Zvi and Elaad, 2018). Such self-assessed dishonesty and lying may be influenced by various internal and external factors, as human perception is essentially biased (e.g., Dror and Murrie, 2018). Specifically, estimates about lying may not be sound and, therefore, one should treat reported lying behavior cautiously. Several studies linked narcissism with actual lying (e.g., Elaad and Zvi, 2019; Elaad et al., 2020). It seems that narcissistic persons lie more than average.

Finally, many studies refer to narcissism as a cohesive entity and examine the construct using global measures. However, it is possible to measure narcissism at the facet level (Ackerman et al., 2011). For example, Ackerman et al. (2011) offered three narcissistic subscales: Leadership/Authority, which depicts feelings of superiority and desire for power, also being considered as an adaptive form of narcissism; Entitlement/Exploitativeness, which captures entitled beliefs and exploitative behaviors, being viewed as maladaptive and even as “socially toxic” narcissism; and Grandiose Exhibitionism, which describes self-importance and exhibitionism; and also seems to represent maladaptive narcissism, although not as maladaptive as Entitlement/Exploitativeness. We will view deception and belief in the present study with each of these dimensions.

#### Self-Assessed Abilities to Tell and Detect Lies

The way people judge their skills is vital because such self-assessed abilities may influence cognition, behavior, and emotions (see Bandura’s self-efficacy theory, 1977). Bandura defined *self-efficacy* as one’s belief in one’s ability to accomplish one’s goals. In the context of lying, self-efficacy beliefs are manifested as self-assessed lie-telling and lie-detecting abilities.

Studies have shown that the ability to tell lies convincingly was rated no better than average (Ekman and O’Sullivan, 1991; Elaad, 2003). The association with honesty explains the relatively low lie-telling ability assessment. In addition, lie-telling competence correlated positively with narcissism (Zvi and Elaad, 2018; Elaad and Zvi, 2019; Elaad et al., 2020) and correlated negatively with religiosity (Elaad, 2018b).

In contrast, people rate their lie-detection abilities higher than average (Elaad, 2018a). Self-assessments of lie-detection abilities are biased because they are inconsistent with actual lie detection performance, as described in a meta-analysis by Bond and DePaulo (2008). Bond and DePaulo indicated that successful lie detection is slightly better than chance. The assumption that most communications are truthful and if not, they can be easily exposed explains the lie-detection bias. The bias is also relevant to the unwillingness to believe that others can easily deceive them. A meta-analysis (Elaad, 2018a) supported this self-assessed lie-telling and lie detection tendencies. When these self-assessed abilities were associated with actual behavior, higher self-assessed lie-telling ability scores were related to reports of frequent lying (Zvi and Elaad, 2018) and with real deception (Elaad and Zvi, 2019; Elaad et al., 2020). There are no reports about an association between lie-detection ability assessments and the correct detection rate of lies.

#### Rational-Experiential Differences in Lying

The Rational-Experiential Inventory (REI), developed by Pacini and Epstein (1999), was used in Experiment 1. Pacini and Epstein (1999) showed that rational scale scores were associated with positive adjustment (low neuroticism, high ego strength, and enhanced self-esteem) and a sense of self-control and direction in one’s life. In addition, when examined against the Big-Five personality dimensions, the scale was positively associated with openness to experience and conscientiousness. The experiential REI scores were associated with the trust of others, interpersonal relationships, and emotional expressivity. The authors further indicated that the scale is positively associated with extraversion, agreeableness, and the ability to express emotions. Conversely, the scale is negatively related to categorical thinking and intolerance.

The REI was incorporated in the present study because it may be relevant to lying and believing. For example, Gino and Ariely (2012) studied the creative personality and explained the tendency of that personality to lie by their ability to justify their unethical behavior. Similarly, we propose that an experiential mindset promotes people’s ability to justify their unethical behavior, which leads to more lying.

Further, Pacini and Epstein (1999) associated the experiential REI scores with extraversion. Specifically, extroverts (positive emotions, assertiveness, sociability, tendency to seek stimulation with others, and talkativeness) are drawn to social life and therefore have more opportunities to lie. Continuing this line of reasoning, Kashy and DePaulo (1996) reported that people involved in social life tend to lie more than people with fewer social opportunities. Barrick and Mount (1991) noted that extroverts perceive themselves as good persuaders. Elaad and Reizer (2015) demonstrated that extroverts rate their lie-telling ability higher than introverts. Therefore, high experiential scorers, who prefer intuitive activities, will lie in the current deception game more than low scorers. Pacini and Epstein (1999) also associated experiential REI scores with trust. Therefore, we hypothesized that high experiential REI scorers would believe senders more than low experiential scorers.

Pacini and Epstein (1999) associated rational REI scale scores with openness to experience (intellectual curiosity, independent thinking, creativity, and preference for novelty and variety) and conscientiousness (self-discipline, act dutifully, focus on achievement). Elaad and Reizer (2015) found a positive association between openness to experience and lie-telling ability assessments and a negative association between conscientiousness and lie-telling ability assessments. Therefore, no hypothesis about the association between rational REI scale scores and lies in the current deception game is forwarded.

Hypotheses made for the first experiment:

1. The competitive men will win the game by frequent lies. But, on the other hand, the less competitive women will stick to the truth bias irrespective of their earned points.
2. The tendency to maintain relationships leads women to believe the sender more than men.
3. Men’s chivalry and women’s solidarity urge participants to lie less to women than to men partners.
3. We expect frequent truth-tellers among senders and recurrent belief among receivers.
4. Narcissistic features, enhanced lie-telling ability assessments, and higher experiential REI scores may explain frequent lying in the sender-receiver deception game.

## Materials and Methods

### Statistical Power and Participants

The present sample consisted of 156 participants from the local community (91 women) with a mean age of 30.9 years (SD = 11.5 years). All participants volunteered to participate in the study and gave their informed consent. The sample size was determined by a G*Power analysis (Faul et al., 2009) which showed that a sample of 111 participants would be appropriate for a study that uses power (1-β) >0.95, and α = 0.05, to detect a medium effect size (*f* = 0.30). We extracted the effect size from a study that correlated narcissistic features, reported lying frequency, and self-assessed lie- and truth-related abilities (Zvi and Elaad, 2018).

### Materials

#### Lie- and Truth Ability Assessment Scale

Participants completed the 16-item Lie-Truth Ability Assessment Scale (LTAAS: Zvi and Elaad, 2018). The items are about four communication abilities: the ability to detect lies (e.g., *In comparison with other people, how would you rate your ability to detect lies?*)*;* the ability to tell lies convincingly (e.g., *In comparison with other people, how would you rate your ability at lying to your peers without getting caught?*); the ability to believe the truths of other people (*e.g., In comparison with your close acquaintances, how good are you at trusting others?*); and the ability to be believed when telling the truth (*e.g., Relative to the average person, how good are you at convincing people to believe you when you are telling the truth?*). Abilities are rated relative to specific others or an average person. The rating scale ranges from 0 (*much worse than others*) to 100 (*much better than others*), with 50 (*as good as others*) supplying a mid-point anchor.

#### Narcissistic Personality Inventory

We used the 40-item Narcissistic Personality Inventory (NPI; Raskin and Hall, 1979; Raskin and Terry, 1988) in the present study. Each item presents a statement such as: *“I am an extraordinary person*; *I find it easy to manipulate people*; and *I like to look at myself in the mirror.”* Participants rated the accuracy of each statement on a 5-point scale with the following definitions: 1 (*not at all true);* 2 (*slightly true*); 3 (*Mediumly true*); 4 (*Largely true*); and 5 (*very much true*). In addition to the NPI global score, which ranges between 40 and 200, we measured narcissism at a facet level (Ackerman et al., 2011). Therefore, we included in the current analysis Ackerman’s three subscales: Leadership/Authority (11 items), Grandiose Exhibitionism (10 items), and Entitlement/Exploitativeness (4 items).

#### Rational-Experiential Inventory

The short 24-items REI is a self-report inventory used by respondents to assess their rational and experiential thinking styles on a 5-point scale ranging from 1 (*not at all true)* to 5 (*very much true*). The rational REI subscale (12 items) measures propensity to analytic thinking and preference of cognitive activities. The experiential subscale (12 items) measures involvement and preference for intuitive activities and experiential-intuitive thinking. Pacini and Epstein (1999) presented support for the reliability and validity of the REI. They demonstrated that individual differences in rational and experiential processing are independent, showing the orthogonal nature of the two-factor structure. Therefore, these are two separate information processing systems.

The Hebrew version (Ayal et al., 2011) of the short 24-items REI was used in the present study. Examples of rational REI statements are: *“I prefer complex problems to simple problems”; “I have a logical mind*.” Examples of experiential statements are: *“I do not have a very good sense of intuition”;* “*I hardly ever go wrong when I listen to my deepest gut feelings to find an answer.”* Cronbach’s alpha coefficients computed for the Hebrew version of the REI were 0.88 and 0.87 for the rational and experiential scales, respectively (Ayal et al., 2011). The authors further showed that the correlation between the two subscales is small and insignificant.

### Procedure

We conducted the present study according to all established ethical guidelines, and the ethics committee of Ariel University approved the study. A female experimenter approached the participants individually and asked them if they were willing to participate in an experiment designed to learn more about lying. First, the experimenter asked participants who gave their oral consent to sign a consent form indicating their agreement to participate in the study. The consent form stated that participants were guaranteed anonymity and could terminate their participation in the study at any time without penalty. Next, the experimenter invited the participants to complete the questionnaires. There was no time limit for completing the questionnaires.

The experimenter conducted the behavioral part of the experiment in pairs. The sender’s role participant received a small box containing one white and one black ball. The sender hid the balls from a partner who served as the receiver. The task of the sender was to pick one ball at choice, hide it in their fist and convince the partner that the picked ball is white. The receiver decided whether to believe the sender or not and indicated it on a form hidden from the sender. Meanwhile, the sender wrote on their form (hidden from the receiver) the color of the selected ball. The goal was to win the game by collecting more points than the other participant in each of the twenty trials of the game. After 10 periods, participants changed roles, and the game continued for another 10 practices. Senders were free to choose any ball, but they had to choose a white ball and a black ball at least once.

Points were distributed according to four possible outcomes:

a. The sender picked a white ball and convinced the recipient to believe them. In that case, the sender receives one point as a token for being believed, and the recipient receives one point for correctly identifying the message as truthful.
b. The sender picked a white ball but was unsuccessful in convincing the recipient to believe them. For this result, the sender received no point because they failed to convince the recipient. The recipient received no point either since they failed to identify the message as truthful.
c. The sender picked a black ball and falsely convinced the recipient that they picked a white ball. For this result, the sender received one point for convincing the recipient that they were telling the truth. The recipient gets no reward because they failed to detect that the sender was lying.
d. The sender picked a black ball and failed to convince the recipient that the ball hidden in the fists was white. The sender received no reward since they failed to convince the recipient with their lies. The recipient received one point for detecting the sender’s lie.

The sender’s payoff matrix is neutral, and senders are equally rewarded by both choices (expectancy of 0.5 points for hiding a black or a white ball). Similarly, the receiver’s payoff matrix is neutral (expectancy of 0.5 points for both believing and disbelieving).

The familiarity between participants in each dyad can affect the prevalence of lies and disbeliefs. To control for a possible familiarity effect, the experimenter asked participants to indicate how familiar they were with their partner and assess how their partner sees their relations. Finally, the experimenter summed up the points collected by each participant and announced the winner. Then, participants were thanked and debriefed about the purpose of the study.

## Results

### Manipulation Check: Familiarity Effects

Participants indicated how they and their partner perceived their relations to test possible mediating familiarity effects. They gave answers on a 7-point scale ranging from 1, not at all acquainted, to seven very closely acquainted. The mean score for their acquaintance question was 2.13 (SD = 1.75), and the mean score for the partner’s acquaintance question was 2.16 (SD = 1.77). Thus, the obtained scores are low, suggesting that familiarity between partners in the game does not affect the results in the present research.

### Number of Lies and Disbeliefs

Due to the truth-telling bias, participants in the sender’s role would lie less than expected by chance and believe the sender more than expected when performing as receivers. Therefore, the total number of lies and disbeliefs were computed and averaged for participants. The means appear in Table 1. We used a one-sample *t*-test to compare the total lying and disbelieving scores with a chance expectancy of 5.0. Results for lies show that *t*_(155)_ = −4.32, *p* < 0.001, *d* = 0.35, indicating that the number of lies is significantly smaller than that expected by chance. On the other hand, we found no significant results for the number of disbeliefs, *t*_(155)_ = −0.28.

**TABLE 1:** Mean frequencies (and SDs) of lies (sender) and disbeliefs (receiver) separated for males and females.

	*N*	Sender’s lies	Receiver’s disbeliefs
Male	65	4.71 (2.11)	5.17 (1.04)
Female	91	4.07 (1.74)	4.92 (1.19)
Across	156	4.33 (1.93)	5.03 (1.13)

*Out of 10.*

### Gender Differences

We hypothesized that men would lie more than women and would believe the sender less than women when in the role of the receiver. Therefore, the mean number of lies and the mean number of disbeliefs were computed separately for men and women and appear in Table 1.

We conducted A 2 × 2 ANOVA with one between-subject factor, gender (men and women), and one within-subject factor, role (sender’s lies and receiver’s disbeliefs) on the mean frequencies in Table 1. A significant role effect, *F*_(1,154)_ = 13.2, *p* < 0.001, η_p_^2^ = 0.079, emerged, indicating that the number of disbeliefs was larger than the number of lies. A significant gender effect, *F*_(1,154)_ = 6.2, *p* = 0.014, η_p_^2^ = 0.039, shows that men tended to lie more than women when in the role of the sender, *t*_(153)_ = 2.07, *p* = 0.04, *d* = 0.34. However, there was no significant belief difference, *t*_(153)_ = 1.36. Finally, the interaction effect was not significant.

The following question is whether these gender differences result from sender-receiver gender dyads? Specifically, would the dyadic gender composition affect frequent lying and disbelieving? Muehlheusser et al. (2015) provided some indications. They grouped participants in pairs and showed more lying in men and mixed groups than in women groups. However, these participants cooperated while participants in the present dyads competed. Therefore, we gathered participants into three gender dyads: Two uniform dyads (two men or two women) and one mixed-gender dyad (one man and one woman). Table 2 presents frequencies of lies and disbeliefs computed for each of the three dyads.

**TABLE 2:** Frequencies of lies (senders) and disbeliefs (receivers) in two uniform and one mixed-gender game dyads.

	*N*	Lies	Disbeliefs
Dyads			
Male/Male	32	4.94 (2.26)	5.16 (1.14)
Male/Female	66	4.30 (2.10)	5.02 (1.18)
Female/Female	58	4.03 (1.41)	4.97 (1.08)

*Out of 10.*

A 2 × 3 ANOVA, two levels of role (senders lies and receivers disbeliefs), and three levels of dyads (men, women, and a mixed-gender dyad), was performed on the mean frequencies presented in Table 2. Results show a significant main effect for role, *F*_(1,153)_ = 10.9, *p* = 0.001, η_p_^2^ = 0.067, indicating that disbelieved responses were more frequent than lies. Table 2 shows that this is true for the three dyads. Although the dyad effect was not significant, *F*_(1,153)_ = 2.5, *p* = 0.082, η_p_^2^ = 0.032, Table 2 shows a gradual decrease in lies and disbeliefs from men dyads through mixed to women dyads. We found no significant interaction effect. A closer inspection of the dyads reveals a significant difference in lying between men and women dyads, *t*_(88)_ = 2.35, *p* = 0.021, *d* = 0.52. Men dyads showed more lying than women dyads. A comparison between the number of lies told by men to fellow men (men dyad) and by men to women in the mixed dyad (M = 4.48, SD = 1.97) reveals no significant difference [*t*_(63)_ = 0.88]. Similarly, lies told by women to female partners (women dyad) are no more or less frequent than lies told by women to men in the mixed dyad (*M* = 4.12, SD = 2.23), *t*_(89)_ = 0.24. We found no significant gender effect for disbeliefs.

### Generating a Performance Index for Production and Detection of Lies and Truths

We generated an individual performance index for every participant and each behavior (production and detection of lies and truths). To this end, we computed the difference between successful and unsuccessful activities. Finally, we averaged the performance indexes across participants and presented them in Table 3.

**TABLE 3:** Means (and SDs) of performance indexes separated for production and detection activities and gender.

	*N*	Production			Detection		
		Lies	Truths	Across	Lies	Truths	Across
Male	65	0.98 (2.03)	0.34 (2.25)	0.66 (1.58)	−0.62 (2.59)	0.58 (2.87)	−0.02 (1.66)
Female	91	−0.02 (2.30)	0.02 (2.82)	0.00 (1.31)	−0.92 (2.33)	0.94 (2.43)	0.01 (1.63)
Across	156	0.40 (2.24)	0.15 (2.15)	0.28 (1.46)	−0.79 (2.44)	0.79 (2.62)	0.00 (3.28)

*A minus sign indicates that failures are more frequent than successes.*

We separated the production and detection indexes in Table 3 and analyzed them separately. A 2 × 2 ANOVA with one between-subject factor, gender (men and women), and one within-subject factor, production (lies and truths), was performed on the lying and truth-telling production indexes. A significant gender effect emerged, *F*_(1,154)_ = 8.12, *p* = 0.005, η_p_^2^ = 0.05. A closer inspection of the results reveals that men were more convincing than women at telling lies, *t*_(154)_ = 2.83, *p* = 0.005, *d* = 0.46, but not at telling truths, *t*_(154)_ = 0.91. We found no production difference between lie-telling and truth-telling and no significant interaction effect.

We performed a similar 2 × 2 ANOVA on the detection indexes, with gender as the between-subject factor and detection as the within-subject factor. A significant detection effect emerged, *F*_(1,154)_ = 23.97, *p* < 0.001, η_p_^2^ = 0.135, indicating that participants were more efficient in detecting truths than lies. We conducted separate matched sample *t*-tests for men and women and found that both complied with the truth detection dominance, men, *t*_(64)_ = 2.23, *p* = 0.029, and women, *t*_(90)_ = 5.13, *p* < 0.001. We did not observe gender or interaction effects for the detection indexes.

### Self-Assessed Lie- and Truth Related Abilities

The LTAAS (Zvi and Elaad, 2018) was used to examine four self-assessed abilities: tell-lies, tell-truths, detect-lies, and detect-truths (each ability comprised four items in the questionnaire). We averaged the four items for each assessed ability and the results, together with the corresponding Cronbach’s alpha reliability scores, appear in Table 4.

**TABLE 4:** Means (and SDs), confidence intervals, and reliability scores separated for the four self-assessed abilities.

	Mean	SD	95% CI	Cronbach’s α
**LTAAS**				
Tell lies	5.45	(2.08)	[5.12, 5.77]	0.91 (*N* = 4)
Detect lies	6.13	(1.84)	[5.83, 6.42]	0.92 (*N* = 4)
Tell truth	6.57	(1.58)	[6.32, 6.82]	0.81 (*N* = 4)
Detect truth	6.78	(1.77)	[6.50, 7.06]	0.89 (*N* = 4)

*Out of 10. N = 156. CI–Confidence Interval is based on the standard error.*

Table 4 shows that all ability assessments are higher than the middle point 5. Since the middle point stands for the assessed ability of the average person, participants attributed higher than average lie and truth communication abilities to themselves. Specifically, all ability assessments are biased. Table 4 further shows that the measurement of all four assessments was reliable. We examined potential differences between the ability assessments using repeated measures one-way ANOVA. While correcting for the linearity assumption (ε = 0.817), a significant ability effect emerged, *F*_(2.45,465)_ = 29.5, *p* < 0.001, η_p_^2^ = 0.16. Results point at differences in the assessment of the four abilities. To further examine these differences, *a priori* Helmert contrasts were applied. The first contrast compared the mean rating of lie-telling, which was predicted to generate the lowest ability assessment, with the mean rating of the other abilities. A significant difference emerged, *F*_(1,155)_ = 169.8, *p* < 0.001. The second contrast compared the mean rating of the ability to detect lies with the mean rating of the two truth-related abilities. A significant difference emerged, *F*_(1,155)_ = 46.9, *p* < 0.001. Results indicated that, as expected, the truth-related abilities were assessed higher than the lie-detecting ability. Finally, the third contrast compared the mean ratings of truth-telling and truth-detection abilities. The difference was not significant.

Gender differences in lie- and truth related ability assessments were also considered. It was found that men rated their lie-telling ability (Mean = 6.06, SD = 2.25), significantly higher than women (Mean = 5.01, SD = 1.84), *t*_(154)_ = 3.21, *p* = 0.002, *d* = 0.52. Similarly, men rated their truth telling ability (Mean = 6.89, SD = 1.62), significantly higher than women (Mean = 6.34, SD = 1.51), *t*_(154)_ = 2.18, *p* = 0.031, *d* = 0.35. No significant gender differences emerged for detection ability assessments.

Finally, we examined self-assessed lie- and truth-related abilities for their informative value as predictors of the number of lies, truths, beliefs, and disbeliefs in the game. We performed four linear regressions where the production and detection behaviors were dependent variables, and the equivalent self-assessed abilities were predictors. Results indicated that self-assessed lie telling ability predicted frequent lie-telling in the game, B = 0.299, β = 0.323, *t* = 4.23, *p* < 0.001, which explains 10.4% of the variance. The three other ability assessments failed to predict the corresponding behaviors. Specifically, high self-assessed lie-detection ability failed to predict a higher disbelief rate in the game. High self-assessed truth detection ability did not predict frequent believing responses. Finally, the high self-assessed truth-telling ability could not predict the truth bias or frequent presentation of white balls.

We employed a similar procedure for each performance index. We found insignificant linear regression predictions for performance indexes from the related self-assessed ability scores. Namely, the self-assessed lie-detection ability did not predict lie-detection performance. Likewise, the self-assessed lie-telling ability could not predict the lie-telling performance index and the perceived truth-telling and truth detecting abilities failed to predict the respective truth indexes.

### Narcissism

We computed narcissistic statistics for men and women (Table 5). Table 5 shows that the reliability of the 40 NPI items was very high. The reliability of the NPI subscales was also extensive. Table 5 presents significant gender differences in global narcissistic scores and the three narcissistic subscales where men show higher narcissistic tendencies than women.

**TABLE 5:** Means and SDs of narcissistic features separated for male and female participants.

	Mean	SD	Cronbach’sα	Gender differences *t*_(154)_
**Narcissism**	**3.16**	**0.86**	**0.97**	
Male	3.48	0.99		
Female	2.93	0.67		4.15**
**Leadership/Authority**	**3.22**	**0.95**	**0.87**	
Male	3.51	1.03		
Female	3.02	0.83		3.28**
**Grandiose Exhibitionism**	**3.01**	**0.96**	**0.90**	
Male	3.29	1.10		
Female	2.81	0.78		3.19*
**Entitlement/Exploitativeness**	**3.09**	**0.94**	**0.7**	
Male	3.47	1.08		
Female	2.82	0.71		4.52**

Males = 65, Females = 91. *p < 0.01, **p < 0.001. Bold values describe the entire sample.

We performed a linear regression analysis to examine the prediction of frequent lying by narcissism. The number of deceptions in the ten-trial session was entered as the dependent variable and the global narcissism score as the independent variable. The analysis indicated that the predicted lying frequency is significant, *B* = 0.698, β = 0.311, *t* = 4.07, *p* < 0.001, and narcissism accounted for 9.7% of the frequent lying variance. We added a hierarchical regression model to predict lying frequency by each of the three subscales of narcissism. We obtained no significant results.

We conducted a similar analysis for the number of disbelieved responses by the receiver in the 10 periods of the game. Again, the global narcissism score and the three subscales failed to predict frequent disbelief.

Next, we examined narcissism as a predictor of the performance indexes. The linear regression model for predicting the truth-telling performance index from the global narcissism score was significant, *B* = 0.62, β = 0.25, *t* = 3.16, *p* = 0.002, and accounted for 6.1% of the index variance. Specifically, receivers believed truthful senders with higher narcissistic records more than truthful senders with lower narcissistic scores. We obtained similar significant results with each of the subscales. Specifically, the truth-telling performance was predicted by Leadership/Authority scores, *B* = 0.55, β = 0.215, *t* = 2.73, *p* = 0.007; Grandiose Exhibitionism scores, *B* = 0.48, β = 0.22, *t* = 2.79, *p* = 0.006; and Entitlement/Exploitativeness scores, *B* = 1.76, β = 0.325, *t* = 4.26, *p* < 0.001. The accounted variances were: 4.6, 4.8, and 10.6% respectively. The narcissism score and the three subscales failed to predict the performance index of lie-telling, lie-detection, and truth detection.

### Rational-Experiential Inventory Scales

Table 6 displays descriptive REI statistics computed for men and women. First, internal consistency for both the rational and experiential scales was adequate. Next, the correlation between the two rankings was non-significant, *r*_(156)_ = −0.137, which confirms the independence of the scales.

**TABLE 6:** Means (and SDs) of rational and experiential rational-experiential inventory (REI) scales separated for male and female participants.

	*N*	Rational scale	Experiential scale
Male	65	3.80 (0.57)	4.13 (0.67)
Female	91	3.73 (0.49)	3.96 (0.52)
Across Gender	156	3.76 (0.59)	4.03 (0.53)
Cronbach’s α		0.74	0.83

Table 6 shows men tendency to be more experiential than women. Using a one-tailed independent-sample t-test indicate, that the experiential difference is significant, *t*_(154)_ = 1.82, *p* = 0.036, *d* = 0.29, and gender differences in the experiential scale scores exists. The rational scale did not show a significant gender difference.

We applied a hierarchical multiple regression analysis on the number of lies in the sender’s ten-trials game. The two REI scale scores were entered as independent variables. Results for the experiential scale, *B* = 0.821, β = 0.252, *t* = 3.22, *p* = 0.002, confirmed the hypothesis that higher experiential scores predicted more lying. Results for the rational scale failed to predict lying frequency. Both scales were unable to predict frequent disbelief in the role of the receiver. We performed a hierarchical regression model for predicting the truth-telling performance index from the two REI subscales. Results show a significant prediction for the experiential scale, *B* = 0.62, β = 0.17, *t* = 2.14, *p* = 0.034, which accounted for 5.3% of the variance. The prediction of the rational scale was insignificant. We conducted similar regressions on the lie-telling, lie detection, and truth detection indexes. In all these cases, we obtained insignificant results.

## Discussion

A meta-analysis that examined gender differences in lying (Capraro, 2018) indicated that men are significantly more likely than women to tell black lies. However, many of the studies in Capraro (2018) analysis used a monetary incentive to guide participant’s actions. Therefore, we cautioned against a possible moderation effect regarding men’s sensitivity to monetary gains and losses and suggested replacing money with game points. Still, we hypothesized that men would lie more to partners than women.

We also monitored other external moderators. Hence, we used a local community sample instead of the typical student sample that may lie more (Abeler et al., 2014; Fosgaard et al., 2018).

The present results, obtained in a face-to-face deception game, support the hypothesis and disaffirm other accounts that found no gender differences in lying (e.g., Childs, 2012). We explained the present results by observing that men rate their lie-telling ability higher than women. Therefore, the mindset that they are able liars may have guided their choices in the game, and men selected to lie more than women.

Similarly, results show that men attribute more narcissistic features to themselves than women. The present results further show that narcissistic qualities predicted more frequent lying, which is in line with previous results that also associated narcissism with deception (e.g., Elaad and Zvi, 2019; Elaad et al., 2020). Hence, narcissism may partly explain the tendency of men to lie more than women in the present deception game.

Finally, men were more persuasive lie tellers than women, which may be associated with their mindset of being competent liars justifying the frequent use of lies. On the other hand, women tended to adhere to the truth-telling bias. Still, the mixed results about men and women lies (e.g., Dreber and Johannesson, 2008; Childs, 2012) suggest that one should be cautious when considering gender differences in lying.

Across gender, self-assessed lie-telling ability scores, higher experiential scores, and higher narcissism scores predicted frequent lying. Furthermore, narcissism and the experiential REI scale predicted the truth-telling performance index. Namely, truth-tellers who scored high on experiential thinking and narcissism were more persuasive than their low-scoring counterparts. The current three scales have an explanatory value in clarifying the production of lies and truths.

As expected, we observed more truth-telling than lying among senders. Thus, the following question is would women stick to the truth bias even when the payoff matrix change and encourage lying and disbelief? We designed Experiment 2 to examine this question.

## Experiment 2

The second experiment examines how men and women change their behavior when applying a payoff matrix that encourages lies and disbelief in a sender-receiver deception game. The second experiment is a constructive replication of the first experiment, with the same gender and self-assessed lie-telling ability hypotheses on deception and disbelief. In addition, we made the following hypothesis:

5. Higher incentives for lying and disbelieving will be associated with more deceit and doubt than in Experiment 1.

## Methods

### Statistical Power and Participants

Experiment 1 reported a strong gender difference in the self-assessed lie telling ability. Further, high lie telling ratings predicted frequent lie-telling in the deception game. Hence, it is not necessary to repeat the large sample size of the first experiment. Instead, we used a G*Power analysis that designated 97 participants to a study that uses power (1-β) > 0.95, and α = 0.05, to detect a medium effect size (*f* = 0.32). The present sample consisted of 100 participants from the local community (40 women) with a mean age of 24.0 years (SD = 7.1) who volunteered to participate in a study on lying and lie detection. Sixty-four participants were secular, 18 traditional, and 19 religious. Participants completed a consent form that promised anonymity and indicated that they were entitled to terminate their participation in the study without penalty.

### Procedure (short)

Participants who consented to participate in the deception game (described in Experiment 1) completed the LTAAS questionnaire individually. Participants were assigned the role of a sender and the role of a receiver (Both of the same gender–man vs. man and woman vs. woman). They changed positions after 10 trials. Upon completion, they were debriefed about the purpose of the study.

The payoff matrix for senders and receivers were as follows:

a. When senders picked a white ball and convinced receivers to believe them, senders received one point for being believed, and receivers got one point for correctly identifying the message as truthful.
b. When senders picked a white ball but were disbelieved, they lost one point as a penalty for failing to convince the receiver of their truthfulness. The receiver got no points.
c. When senders hid a black ball in their fist and convinced the receiver to believe them that the ball was white, senders received one point for their compelling message. Receivers lost one point as a penalty for not detecting the lie.
d. When senders chose a black ball and failed to convince the recipient that the ball was white, senders received no point, and receivers added one point to their pack for detecting the sender’s lie.

The sender’s payoff matrix encourages senders to lie (expectancy of 0.5 points for hiding a black ball compared to 0 points for hiding a white ball). Similarly, the receiver’s payoff matrix encourages receivers not to believe (expectancy of 0.5 points for disbelieving compared to expectancy of 0 points for believing).

Senders did not reveal the color of the chosen ball, and receivers did not inform their decision at any point in the experiment. Instead, both participants prescribed their choices on a form hidden from the other participant.

## Results

### Familiarity Effects

To examine how familiar the interacting pairs were, we asked participants to indicate how close they were to their partner and how close their partner would feel acquainted with them. Scales ranged from (1) not at all acquainted to (7) very much. The average of the two answers defined familiarity. The mean familiarity score was 1.74 (SD = 1.00) out of 7, indicating that the interacting pairs were not acquainted.

### Number of Lies and Disbeliefs

As in Experiment 1, we computed the number of lies and disbeliefs. Due to the payoff matrix, which encouraged lying and disbelief, we hypothesized that participants in the sender’s role would increase their lying frequency. In the receiver’s position, participants will disbelieve more than expected by chance. The average number of lies and disbeliefs appear in Table 7. We used a one-sample t-test to compare mean lying and mean disbelieving scores in Table 7 with a chance expectancy of 5.0. Disbeliefs show that *t*_(99)_ = 2.54, *p* = 0.013, *d* = 0.25, indicating that the number of disbeliefs is significantly larger than that expected by chance. However, the effect size is small. We obtained no similar results for the number of lies, *t*_(99)_ = 0.98. Still, another way to look at the motivation effect is to compare the present results with those of Experiment 1, which applied no motivation to lie or stimulate disbelief. The comparison reveals that lie-telling was more frequent in the present experiment than in the first experiment, *t*_(254)_ = 3.56, *p* < 0.001, *d* = 0.46 (see Tables 1, 7). We obtained similar results for disbelieving, *t*_(254)_ = 2.03, *p* = 0.022, *d* = 0.25.

**TABLE 7:** Mean frequencies (and SDs) of lies (sender) and disbeliefs (receiver) separated for males and females.

	*N*	Sender’s lies	Receiver’s disbeliefs
Male	60	5.52 (1.64)	5.43 (1.53)
Female	40	4.63 (1.46)	5.23 (1.12)
Across	100	5.16 (1.63)	5.35 (1.38)

*Out of 10.*

Table 7 also presents gender differences in frequent lying. A 2 × 2 ANOVA with gender (men, women) as a between-subject factor, and role (sender’s lies and receiver’s disbeliefs) as a within-subject factor, was applied on the mean frequencies in Table 7. Results showed a significant gender effect, *F*_(1,98)_ = 4.71, *p* = 0.032, η_p_^2^ = 0.046, and a significant interaction effect, *F*_(1,98)_ = 4.29, *p* = 0.041, η_p_^2^ = 0.042. No significant role effect was found, *F*_(1,98)_ = 2.45. Specifically, men lie more frequently than women, *t*_(98)_ = 2.78, *p* = 0.007, *d* = 0.57, but there was no significant gender difference in disbelieving, *t*_(98)_ = 0.74. A matched sample *t*-test shows that women disbelieved more than they lied, *t*_(39)_ = 2.42, *p* = 0.02. Men disbelieved and told lies at a similar high frequency.

### Production and Detection Indexes of Lies and Truths

As indicated in Experiment 1, we computed the difference between successful and unsuccessful activities for every participant and each behavior to generate individual performance indexes. Then we averaged the scores across participants and displayed them in Table 8. Note that production and detection indexes are separated.

**TABLE 8:** Means (and SDs) of performance indexes separated for production and detection activities and gender.

	*N*	Production			Detection		
		Lies	Truths	Across	Lies	Truths	Across
Male	65	−1.58 (2.53)	0.72 (1.66)	−0.43 (1.53)	1.67 (2.28)	0.63 (2.15)	1.15 (1.49)
Female	91	−0.98 (2.03)	0.53 (1.87)	−0.23 (1.12)	0.38 (2.44)	1.13 (1.86)	0.75 (1.80)
Across	156	−1.34 (2.35)	0.64 (1.74)	−0.35 (1.38)	1.15 (2.42)	0.83 (2.05)	0.99 (1.54)

*The performance index shows the difference between the number of successful activities and failures. Hence, a minus sign indicates that failures are more frequent than successes.*

A 2 × 2 ANOVA was performed on the production indexes with gender (men, women) as the between-subject factor and production (lies, truths) as the within-subject factor. A significant production effect, *F*_(1,98)_ = 36.73, *p* < 0.001, η_p_^2^ = 0.27, indicated that participants were more persuasive truth-tellers than liars. We found no significant gender or interaction effects.

We applied a second 2 × 2 ANOVA on the detection indexes with gender as the between-subject factor and detection of lies and truths as the within-subject factor. We found no significant gender or detection effects. However, a significant interaction effect emerged, *F*_(1,98)_ = 7.72, *p* = 0.007, η_p_^2^ = 0.07. A closer inspection of the results reveal that men performed better than women as lie-detectors, *t*_(98)_ = 2.70, *p* = 0.008, *d* = 0.55, but no significant gender difference exists when we considered truth detection [t_(98)_ = −1.18). Furthermore, when using a matched sample *t*-test, we found that men are better lie-detectors than truth detectors, *t*_(98)_ = 2.44, *p* = 0.018. Women tend to be better at truth detection than lie detection, but the difference is insignificant [*t*_(98)_ = −1.62].

### The Contribution of Self-Assessed Ability Ratings to Frequent Deception

As in Experiment 1, measurements of all four assessments were reliable, and all ability assessments were rated higher than the middle point 5 (see Table 9). Specifically, participants attributed higher than average lie- and truth-related abilities to themselves. Using a repeated measures one-way ANOVA, and correcting for linearity (ε = 0.825), a significant ability effect was observed, *F*_(2.41,238.6)_ = 31.6, *p* < 0.001, η_p_^2^ = 0.24. Hence, there are differences in the assessment of the various abilities. *A priori* Helmert contrasts were applied to examine these differences. As in Experiment 1, we contrasted the lie-telling ability assessment with the mean rating of the other abilities. A significant difference emerged, *F*_(1,99)_ = 28.1, *p* < 0.001, η_p_^2^ = 0.22. As in Experiment 1, the second contrast compared the mean rating of the ability to detect lies with the mean rating of the two truth-related abilities. The difference is significant, *F*_(1,99)_ = 42.2, *p* < 0.001, η_p_^2^ = 0.30. As expected, we observed a higher assessment of truth-related abilities than the assessment of the lie-detecting ability. The final contrast compared the mean ratings of the abilities to tell and detect truths. Unlike Experiment 1, the difference is significant, *F*_(1,99)_ = 24.0, *p* < 0.001, η_p_^2^ = 0.195. Namely, the truth detection ability was assessed higher than the truth-telling ability.

**TABLE 9:** Statistics of the LTAAS.

	Mean	SD	95% CI	Cronbach’s α
Tell lies	59.6	17.8	[56.0, 63.2]	0.90 (*N* = 4)
Detect lies	61.4	14.9	[58.4, 64.4]	0.91 (*N* = 4)
Tell truth	66.6	10.5	[64.5, 68.7]	0.89 (*N* = 4)
Detect truth	73.3	12.4	[70.8, 75.7]	0.73 (*N* = 4)

*N = 100. CI = Confidence interval based on standard error units.*

Considering gender differences in lie- and truth related ability assessments, it emerged that men assessed their lie-telling ability (Mean = 6.36, SD = 1.69), higher than women (Mean = 5.36, SD = 1.76), *t*_(98)_ = 2.85, *p* = 0.005, *d* = 0.58. No significant gender differences were found for the three other ability assessments.

Four linear regressions were applied to examine if self-assessed lie- and truth-related abilities predict frequent lies, truths, beliefs, and disbeliefs. The self-assessed abilities were predictors, and sums of telling and detecting behaviors served as dependent variables.

Results indicated that low self-assessed lie-detecting ability predicted frequent lie-detecting in the game, *B* = −0.027, β = −0.293, *t* = −3.04, *p* = 0.003. which explains 8.6% of the variance. Specifically, high self-assessed lie-detection ability predicted a lower disbelieving rate in the game. We suggested that people who score high on their lie detection ability assessment are more careful in using disbeliefs than their counterparts who displayed lower self-assessments. The three other ability assessments failed to predict the corresponding behaviors, and we failed to replicate the results of Experiment 1.

## Discussion

Manipulating the payoff matrix to encourage deception and doubt increased the frequency of lie-telling and disbelieving in the second experiment.

As to gender differences, while men lied more frequently than women, there was no significant gender difference in disbelieving. Results support those of Capraro (2018), who suggested that men are more involved in black lies than women.

The production performance index indicated that irrespective of gender, participants performed better as truth-tellers than liars. However, the detection performance index interacted with gender and showed that men were more efficient lie-catchers than women.

As expected, men assessed their lie-telling ability higher than women, but we found no gender differences for the three other ability assessments. Unfortunately, self-assessed lying ability failed to predict frequent lying in the game. Irrespective of gender differences, high self-assessed lie-detection capacity indicated less suspicion in the receiver’s position than low lie-detection ability.

## General Discussion

The first experiment examined gender differences under strict control over many situational incentives to lie. Specifically, we ensured that the payoff matrix does not drive participants to prefer lying and disbelieving. We also removed any monetary reward that could differentially motivate lying. To control reciprocity, we kept secret sender’s and receiver’s decisions. In addition, we ensured that familiarity among participants was minimal. Participants faced each other, but their verbal communication was limited to a sender’s statement about the ball’s color in their fists. Receivers kept silent and only wrote down their decision. In this way, we controlled the flow of talking, which could have influenced lying and disbelieving.

Further, senders were free to decide whether to pick a white or a black ball, given that they choose one ball of each color at least once, and receivers were free to decide whether to believe the sender or not. Next, in some earlier studies, the gender of the partner remained unknown (e.g., Dreber and Johannesson, 2008; Conrads et al., 2013). In these cases, men used selfish lies more often than women. Therefore, we removed the moderating effect by ensuring that the present participants kept eye contact and knew the partner’s gender. Further, most previous deception studies employed students as participants (Gerlach et al., 2019). Since students are typically younger than a more representative sample of the population and have a better cognitive ability to justify their dishonest behavior (Gino and Ariely, 2012; Gerlach et al., 2019), they act more dishonestly (Abeler et al., 2014; Fosgaard et al., 2018). Therefore, we sampled our participants from the local community. Our setting is unique to our knowledge because no other study has paid attention to all these potential moderators.

As expected, we observed frequent truth-telling and believing in the first experiment, and the number of lies was significantly smaller than that expected by chance. We may explain the results by expectations such as the predominant truth-telling bias. It seems that women are more sensitive to the truth-telling bias than men. Still, the number of lies was considerable (43.3%), and we attributed them to internal motivation, including personal tendencies, perceived abilities, and preferred cognitive style.

In the second experiment, the payoff matrix inspired participants to lie more in the sender’s position, believe less in the receiver’s role, and challenge the truth-telling bias. Indeed, results showed more lie-telling and a higher disbelieving rate than in the first experiment.

Still, some differences exist between the samples of the two experiments. First, the participants in the second experiment are younger. Besides participants from the local community, the sample comprised more students and other young people than in the first experiment. Thus, we may attribute some of the increased lying rates to the young age of the participants.

Second, considering the different men and women in the two experiments, the truth-bias explanation may be compromised. Specifically, women comprised 58.3% of the sample in the first experiment, whereas they consisted of only 40% in the second experiment. As men tend to lie more than women, we can attribute the increased lying rate in the second experiment to the more significant men frequency. Still, the second experiment showed a similar increase of disbelieving by men and women, which we attribute to the payoff matrix. Thus, the gender composition in the two experiments has only a marginal effect in explaining the obtained gender differences (Tables 1, 7).

In both experiments, we found significant gender differences in frequent lying. A comprehensive meta-analysis about gender differences in black lies (Capraro, 2018) supports our result. Capraro indicated that men are significantly more likely than women to tell black lies: “males are more selfish than females and more concerned about social efficiency than females; while females are more concerned than males about reaching an equitable distribution of payoffs” (p. 353). Thus, we may suggest that gender differences in lying exist. The first experiment indicated that men’s disposition to lie more than women in a face-to-face deception game derives from men’s enhanced assessment of their lying ability, stronger narcissistic tendencies, and to some extent, men’s experiential thinking style. In particular, the first experiment provides evidence for gender differences in the self-assessed ability to lie persuasively. Men tended to assess the ability higher than women. Self-assessments are related to self-efficacy (Bandura, 1977). Bandura suggested that the way people perceive their skills may provide further information on how such perceptions influence emotions, cognition, and behavior. Feeling that they are capable liars may have driven men choices in the game. They chose to lie more frequently than women who were less convinced about their lying ability and adhered to the truth bias.

Experiment 1 showed that men scored higher on narcissistic features than women, which predicted frequent lying. Results agree with earlier studies that associated narcissism with actual deception (e.g., Elaad and Zvi, 2019; Elaad et al., 2020). Therefore, we suggest that narcissism may explain, in part, the tendency of men to lie more than women. Finally, in the first experiment, higher experiential scores predicted frequent lying, and men tended to show more experiential thinking than women, which explains men’s tendency to lie more.

The second experiment replicated the first experiment’s results for gender differences in frequent lying. Again, we found that men lied more frequently than women, while no significant gender difference exists in believing. Nevertheless, the literature on the association between gender and frequent lying presents some negative results (e.g., Childs, 2012), implying that research on gender and deception should continue to clarify these inconsistencies.

Next, we dealt with successfully telling and detecting lies and truths. To this end, we generated performance indexes based on the difference between successful and unsuccessful production and detection activities. Experiment 1 revealed no significant production difference between lies and truths. As to gender effects, men were more persuasive liars than women. Analyzing the detection indexes reveal agreement with the truth bias. Specifically, participants were more efficient truth detectors than lie detectors, which is true for both men and women. Finally, no significant gender differences exist for the detection indexes.

Experiment 2 failed to replicate these results. Irrespective of gender differences, participants were more persuasive truth-tellers than liars. As to the detection indexes, men performed better as lie detectors than truth detectors and were more efficient lie detectors than women. Motivation differences cannot explain the inconsistent results of the two experiments. We may join Burgoon et al. (2006) conclusion about overstating the extent of sex differences. Nevertheless, the present results recommend caution in interpreting them. Future research should take the responsibility to clarify the inconsistencies, possibly by using larger experimental samples to compensate for the relatively small effect.

In the first experiment, we paid attention to sender-receiver gender dyads. We examined two single-gender dyads (two men or two women) and one inter-gender dyad (one man and one woman) for their effects on lying and believing. Results presented a gradual decrease in lies and disbeliefs from men dyads through mixed dyads to women dyads (Table 2). However, the effect is not significant. Further, the number of lies told by men to fellow men in men dyads was not different from that in mixed dyads. When we examined women’s lies, the lies told to other women were not more frequent than those delivered to men.

In both experiments, we asked participants to complete the LTAAS questionnaire. Results of the LTAAS were in complete agreement in the two experiments, which contributes to the reliability of the questionnaire. In both experiments, men assessed their lie-telling ability higher than women, but unlike the first experiment, lie-telling assessment scores in the second experiment failed to predict frequent lying. The last result may be unusual considering earlier outcomes that showed a consistent association between high self-assessed lying ability, reports of frequent lying (Zvi and Elaad, 2018), and predictions of actual lying in experimental settings (Elaad and Zvi, 2019; Elaad et al., 2020). Thus, confidence in telling lies drives people to increase their use of lies.

As to the LTAAS, earlier studies showed that the ability to tell lies convincingly was rated no better than average (e.g., Elaad, 2003; Elaad, 2018a). The present results showed that participants assessed their lie-telling ability higher than average in both experiments. Despite the high lie-telling ability ratings, gender differences in evaluating the lie-telling ability persist.

The LTAAS provides in addition information about self-assessed lie-detection ability. Inconsistent with the poor actual lie-detection performance (see a meta-analysis by Bond and DePaulo, 2008), people award above-average ratings to their lie-detecting skills (e.g., Elaad, 2018a). We explained the lie-detection bias by people’s tendency to maintain a good impression. People like to believe that their success in detecting lies protects them from being easily deceived by others.

We obtained higher than average lie-detection ratings in the two present experiments. Previous accounts did not associate the lie-detection bias with frequent disbelieving in experimental settings. In this respect, the association between high self-assessed lie-detection ability and low disbelief rate under conditions that encourage disbelief is surprising. Results suggest that people with confidence about correctly detecting lies tend to restrain disbelieving in the experimental setting. We found no such restrain under conditions that stimulated truth-telling and believing. We observed this link for the first time, to the best of our knowledge, and therefore, the result deserves additional research.

Previous research indicated strong bonds between self-assessed lie-telling ability and narcissistic features (Zvi and Elaad, 2018; Elaad et al., 2020). The present results are not different, and the correlation between lie-telling ability assessments and global narcissism scores is very high, *r*_(156)_ = 0.66. Giammarco et al. (2013) further noted that narcissistic individuals believe to be better liars than the average person. Additionally, as with lie-telling assessments, men showed significantly higher global narcissism scores than women. Men scored higher than women also in the three narcissistic subscales: Leadership/Authority, Grandiose Exhibitionism, and Entitlement/Exploitativeness.

Earlier accounts described positive relations between narcissism and reported lying or unethical behavior in everyday life situations (Oliveira and Levine, 2008; Baughman et al., 2014; Azizli et al., 2016). Specifically, the described literature indicates that narcissistic individuals lie more and trust less than average. Thus, predictions about the association between narcissism and frequent lying received support in the present study.

We hypothesized that high REI experiential scores predict frequent lying in the deception game. Results confirmed the hypothesis. Pacini and Epstein (1999) found a positive association between high experiential scores and extraversion. Although we did not examine extraversion in the present study, some aspects of extraversion that were discussed may be appropriate to explain the results. For example, extrovert’s social experience and the opportunities to lie and improve their lying skills drive them to be confident in their lying abilities (Elaad and Reizer, 2015). We found similar associations between confidence in lie-telling and actual lying in the present deception game. In contrast, high REI experiential scorers did not believe senders more than lower experiential scorers. Finally, we found no significant associations between rational REI scores and lie telling frequency or believing frequency.

We examined for the first time the association between high REI experiential scores and frequent lying under the influence of the truth-telling bias. Future research should apply the REI scales under conditions that explicitly encourage lying.

### Limitations and Suggestions for Future Research

When we looked for interaction effects, we lacked power. The overall gender effect is too small to support such interactions with our present sample. Future research interested in possible gender interactions should use a much larger sample.

The present design is limited to low stakes. Participants knew that they were participating in a game, and they would not experience any consequences from their deception in the game. Stakes are essential because lies often entail mental effort, unlike the present low-stakes design (Vrij, 2008). When telling high-stake lies, liars are committed to the lie, must maintain the fake story, remember what they said earlier to whom and under which circumstances, be careful not to contradict themselves, and convey a logical sequence of events. For these reasons, liars experience increased cognitive load, which we spared from the present participants. Future research should compare two stake levels to look for possible effects of cognitive load on preferences for lying or truth-telling in a face-to-face deception game.

## Data Availability Statement

The original contributions presented in the study are included in the article/supplementary material, further inquiries can be directed to the corresponding author.

## Ethics Statement

The studies involving human participants were reviewed and approved by Ariel University Ethics Committee. The participants provided their written informed consent to participate in this study.

## Author Contributions

EE initiated the study, formulated the hypotheses, designed the experiments, supervised all tasks and procedures that research assistances carried out, analyzed the data, and wrote the manuscript. YG-G designed Experiment 1, supervised research assistances who conducted this experiment, analyzed the data of the first experiment, described and discussed the results in a master thesis report. Both authors contributed to the article and approved the submitted version.

## Conflict of Interest

The authors declare that the research was conducted in the absence of any commercial or financial relationships that could be construed as a potential conflict of interest.

## Publisher’s Note

All claims expressed in this article are solely those of the authors and do not necessarily represent those of their affiliated organizations, or those of the publisher, the editors and the reviewers. Any product that may be evaluated in this article, or claim that may be made by its manufacturer, is not guaranteed or endorsed by the publisher.
